# Supplementary material for: In Vivo Flow Cytometry of Extremely Rare Circulating Cells
Source: Sci Rep. 2019 Mar 4;9:3366. doi: 10.1038/s41598-019-40143-2 (PMC6399281; doi:10.1038/s41598-019-40143-2)
Supplement: Supplementary file 1 — Supplementary Information [file 41598_2019_40143_MOESM1_ESM.pdf]

In Vivo Flow Cytometry of Extremely Rare Circulating Cells
Supplementary Information

Xuefei Tan[1], Roshani Patil[2], Peter Bartosik[2], Judith Runnels[3], Charles P. Lin[3] and Mark Niedre[1,2]*

[1]Northeastern University, Department of Electrical and Computer Engineering, Boston, MA, 02115

[2]Northeastern University, Department of Bioengineering, Boston, MA, 02115

[3]Center for Systems Biology and Wellman Center for Photomedicine, Massachusetts General Hospital and Harvard Medical School, Boston, MA, 02114

Supplementary Figures

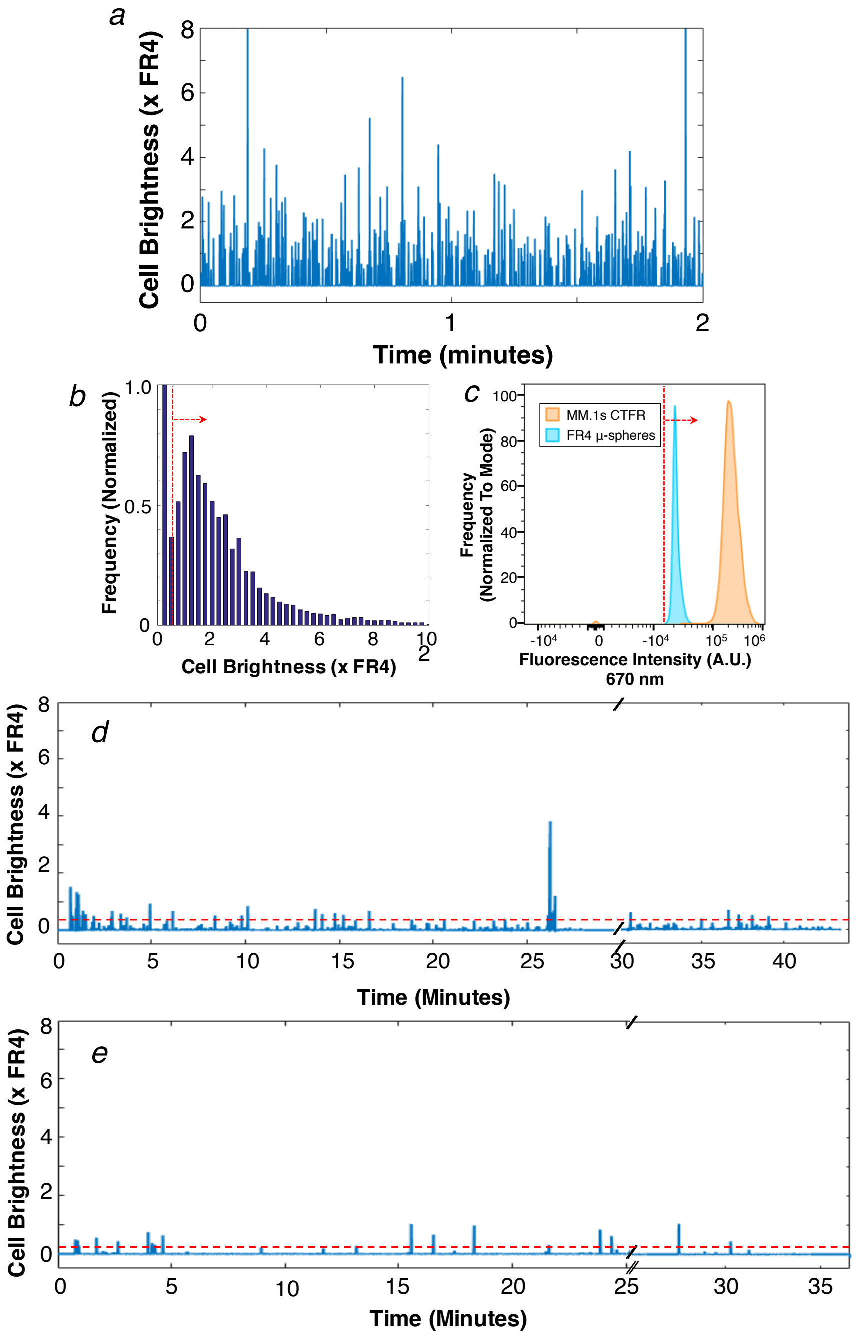

Supplementary Figure S1. *(a) An example DiFC trace of CTFR-labeled MM cells in vitro, normalized to FR4 reference microspheres. (b) Histogram of peak intensities of CTFR- labeled MM cells measured with DiFC, and (c) measured with a flow cytometer. (d,e) Example DiFC data measured from two mouse blood samples. The dotted red lines indicate the counting threshold of 50% of FR4 microspheres. See text for details*

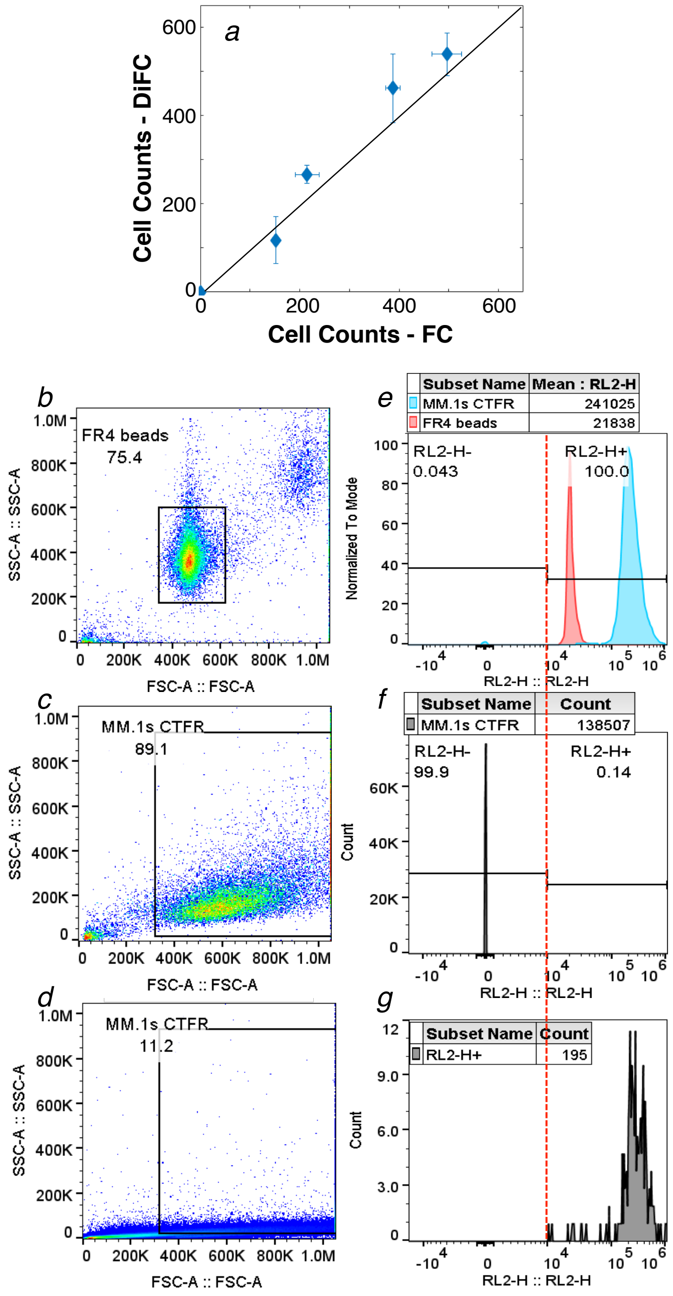

Supplementary Figure S2. *(a) We verified the counting accuracy of DiFC against flow cytometry (FC) for blood samples spiked with CTFR-labeled MM cells. Range bars represent the variability over 3 trials. The FC gating methodology for CTFR is shown: (b) SSC-FSC plot for FR4 reference beads. (c) SSC-FSC plot for CTFR-MM stock cell suspension. (d) SSC-FSC plot for whole blood spiked with approximately 250 CTFR-MM cells. The gate was selected from the CTFR-MM stock suspension in panel (c). (e) Fluorescence (RL2-H, 710/50nm) histogram for FR4 reference beads and CTFR-MM stock suspension, used to select the counting threshold (vertical dotted red line). (f) Fluorescence histogram from the spiked blood sample in the SSC-FSC gate from panel (d). (g) Fluorescence histogram of RL2-H+ cells in the gate.*
